# Impact of Individual Traits, Saturated Fat, and Protein Source on the Gut Microbiome

**DOI:** 10.1128/mBio.01604-18

**Published:** 2018-12-11

**Authors:** Jennifer M. Lang, Calvin Pan, Rita M. Cantor, W. H. Wilson Tang, Jose Carlos Garcia-Garcia, Ira Kurtz, Stanley L. Hazen, Nathalie Bergeron, Ronald M. Krauss, Aldons J. Lusis

**Affiliations:** a Department of Medicine, Division of Cardiology, University of California, Los Angeles, California, USA; b Department of Human Genetics, University of California, Los Angeles, California, USA; c Lerner Research Institute, Cleveland Clinic, Cleveland, Ohio, USA; d Life Sciences TPT, Procter & Gamble, Cincinnati, Ohio, USA; e Department of Medicine, Division of Nephrology, University of California, Los Angeles, California, USA; f Department of Biological and Pharmaceutical Sciences, College of Pharmacy, Touro University California, Vallejo, California, USA; g Children’s Hospital Oakland Research Institute, Oakland, California, USA; h Department of Microbiology, Immunology and Molecular Genetics, University of California, Los Angeles, California, USA; University of Maryland, School of Medicine

**Keywords:** diet, diversity, gut microbiome, personal traits, protein, saturated fat

## Abstract

Interindividual variation in the composition of the human gut microbiome was examined in relation to demographic and anthropometric traits, and to changes in dietary saturated fat intake and protein source. One hundred nine healthy men and women aged 21 to 65, with BMIs of 18 to 36, were randomized, after a two-week baseline diet, to high (15% total energy [E])- or low (7%E)-saturated-fat groups and randomly received three diets (four weeks each) in which the protein source (25%E) was mainly red meat (beef, pork) (12%E), white meat (chicken, turkey) (12%E), and nonmeat sources (nuts, beans, soy) (16%E). Taxonomic characterization using 16S ribosomal DNA was performed on fecal samples collected at each diet completion. Interindividual differences in age, body fat (%), height, ethnicity, sex, and alpha diversity (Shannon) were all significant factors, and most samples clustered by participant in the PCoA ordination. The dietary interventions did not significantly alter the overall microbiome community in ordination space, but there was an effect on taxon abundance levels. Saturated fat had a greater effect than protein source on taxon differential abundance, but protein source had a significant effect once the fat influence was removed. Higher alpha diversity predicted lower beta diversity between the experimental and baseline diets, indicating greater resistance to change in people with higher microbiome diversity. Our results suggest that interindividual differences outweighed the influence of these specific dietary changes on the microbiome and that moderate changes in saturated fat level and protein source correspond to modest changes in the microbiome.

## Abstract (precis)

The microbiome has proven to influence health and disease, but how combinations of external factors affect the microbiome is relatively unknown. Diet can cause changes, but this is usually achieved by altering macronutrient ratios and has not focused on dietary protein source or saturated fat intake levels. In addition, each individual’s unique microbiome profile can be an important factor during studies, and it has even been shown to affect therapeutic outcomes. We show here that the effects of individual differences outweighed the effect of experimental diets and that protein source is less influential than saturated fat level. This suggests that fat and protein composition, separate from macronutrient ratio and carbohydrate composition, is an important consideration in dietary studies.

## INTRODUCTION

Dietary influences are mediated in part by the gut microbes, which consist of hundreds of different bacterial species as well as fungi and viruses. These microbes metabolize certain dietary components, including complex carbohydrates that are otherwise indigestible, and produce hundreds of novel molecules, some of which are absorbed into the circulation and have physiologic or disease-related effects (1–3). One of the complicating factors in assessing the effects of a particular diet is that the individual components can interact in synergistic or antagonistic ways (4–7).

No studies in humans have investigated the interacting effects of dietary protein source and saturated fat level on the microbiome. Many have compared diets differing in ratios of macronutrients (e.g., proteins, fats, carbohydrates) without investigating the effects of specific nutrient groups. Indigestible carbohydrates are considered the primary resource for intestinal microbes (1, 8) and can change the microbiome (9, 10), while proteins are an important nitrogen source (11). Fat is generally considered to be less important to the metabolism of microbes (11), but dissimilar effects of saturated fat and fish oil on the microbiome suggest that there may be an effect through other mechanisms (2, 12) and that fat quality is an important consideration in dietary studies.

Moreover, interindividual differences increase the complexity and uniqueness of the microbiome with factors such as sex (13, 14), age (15, 16), genetics (17), ethnicity (18, 19), obesity (20–22), medications (23, 24), and previous dietary habits (25, 26) being important. It is not uncommon for the unique microbiome profile inherent to the individual to remain during dietary interventions (9, 27), especially short-term ones, because it is difficult to overwhelm historical effects on the microbiome (25). It has also been shown that the response to dietary challenges depends in part on the microbiota that are present in the gut (28–30). Obvious and rapid changes are seen with severe dietary changes (31), but the experimental diets in the present study reflect realistic dietary changes within ranges of what is consumed by Americans (32).

**FIG 1:**
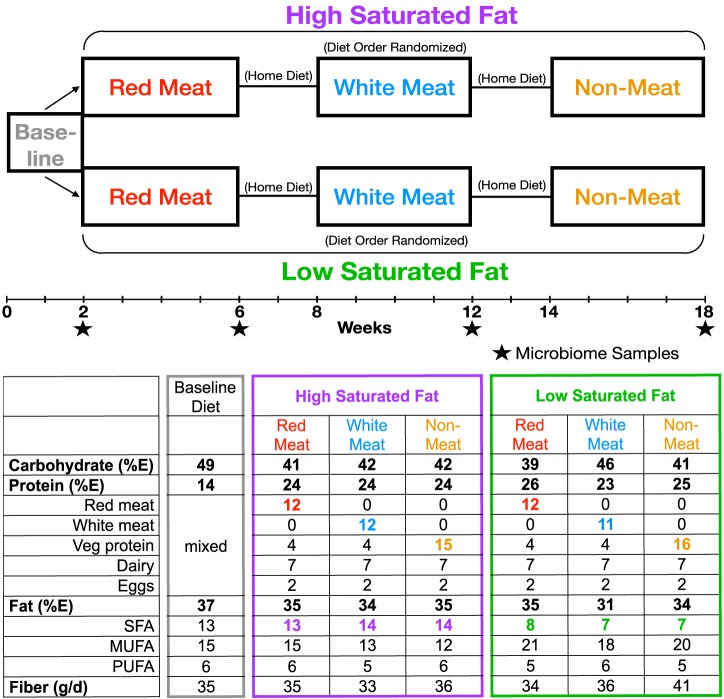
Study design and dietary composition breakdown of APPROACH study. All participants were put on a baseline diet for 2 weeks and then separated into low- and high-saturated-fat groups for the experimental diets. Within the fat group, the protein diets were randomized to create a split-plot design, meaning that participants received all protein treatments but only one fat level. Experimental diets lasted 4 weeks with a 2-week, but up to 7-week, washout period where participants ate their home diet. Levels are based on compositional analysis of 10,460-kJ four-day rotating menus. Protein and fiber were calculated values (Nutrition Data System for Research, University of Minnesota) to include adjustments of compositional analysis of daily menus.

This study aimed to determine the interacting effects of dietary saturated fat level and protein source (beef, chicken, and vegetable) on the microbiome using a randomized controlled human dietary intervention trial (Fig. 1). Samples from 109 healthy men and women who ranged in age (21 to 65 years) and body mass index (18 to 36) were sequenced using the 16S rRNA gene. Four fecal samples were taken from each participant: one initial baseline sample and one each after each of the three experimental diets. Diets were well controlled, as ∼90% of the food was produced and provided by a metabolic kitchen, and participants were responsible only for the purchase of small amounts of fresh produce. We report here the effects of the diets on gut microbiota composition.

## RESULTS

### Four-week dietary interventions minimally change the microbiome.

**FIG 2:**
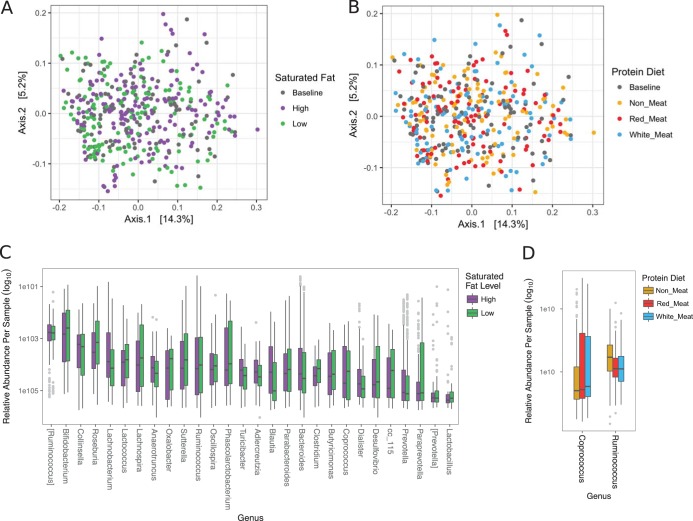
Overall dietary influence on the microbiome. Unweighted UniFrac PCoA data of fecal samples from 109 participants are labeled by (A) saturated fat level and (B) protein diet. Differentially abundant OTUs between (C) saturated fat level and (D) protein diet were determined by DESeq2 using age, sex, ethnicity, and diet order as covariates. Significant OTUs with *P* values (Benjamini-Hochberg corrected) that are described at the genus level are displayed as relative abundance within each diet.

Overall, the dietary interventions caused modest changes in the microbiome. Principal-coordinate analysis with unweighted UniFrac distance displayed no clustering of samples by diet (Fig. 2). Alpha diversity, which is a measurement of how many taxa are present that also takes into account the distribution of the taxa (i.e., are the counts evenly distributed or skewed), trended toward significance comparing saturated fat level (two-way ANOVA, *F *=* *2.97, *P = *0.088) but not dietary protein source (two-way ANOVA, *F *=* *1.27, *P = *0.28), and there was no interaction between the two (two-way ANOVA, *F *=* *0.73, *P = *0.48). Beta diversity represents how much the community changed in comparison to the baseline diet and was calculated as the distance between the experimental diets and the baseline diet for each participant. There were no differences in beta diversity and the *Firmicutes*/*Bacteroidetes* ratios (log_2_).

Saturated fat level was more influential than protein source on taxon abundance. Differential abundance was modeled using a negative binomial distribution that accounted for sex, age, ethnicity, and diet order, and significance was estimated using Wald’s test in the DESeq2 package (33). There were 151 differentially abundant OTUs between low and high saturated fat levels, and 57 were described at the genus level (Fig. 2). Three OTUs were differentially abundant between the various protein diets, and only two were described at the genus level (Fig. 2). To determine the effect of the changing macronutrient ratio from the baseline diet (Fig. 1), the baseline diet was compared to the first experimental diet regardless of protein source. This seemed to have a minimal effect on taxon abundance, as only seven OTUs were determined to be differentially abundant, further highlighting the unexpected result of the effect of saturated fat level on microbial abundance.

### Demographic and anthropometric traits outweigh dietary interventions and are significantly associated with overall microbiome community composition.

**FIG 3:**
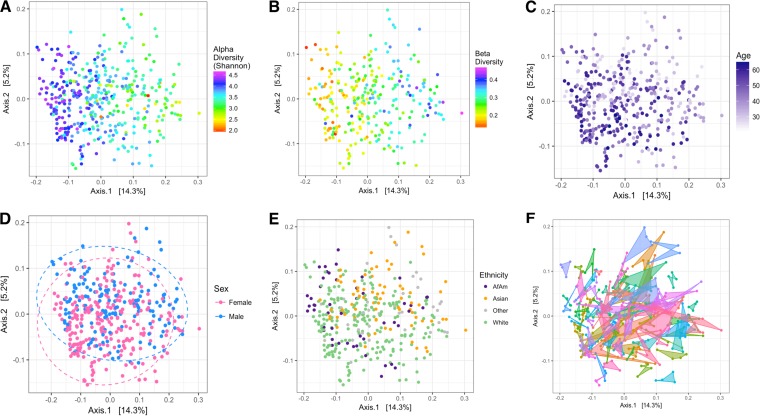
Participant characteristics outweighed dietary interventions and drove overall microbiome community composition. Continuous traits were fit to the ordination as vectors using regression, and (A) alpha diversity (*P < *0.001), (B) beta diversity (*P < *0.001), and (C) age (*P < *0.001) were the most influential. Categorical variables were tested with nonparametric multivariate analysis of variance (PERMANOVA) to determine if variables were clustered significantly differently, and (D) sex (*P < *0.001), (E) ethnicity (*P < *0.001), and (F) participant (*P < *0.001) were significant. Dashed lines for sex represent 95% confidence interval from the centroid of the cluster. Polygons connect all of the samples from one participant.

The strongest influential determinants of microbiome composition and PCoA ordination were traits describing interindividual variation. Measured and calculated variables were fit to the ordination as vectors using regression to determine which were significantly related to the plot sample distribution. Alpha diversity had the strongest association with microbiome composition and PCoA ordination (*r^2^* = 0.48, *P < *0.001) (Fig. 3A), but beta diversity was also highly significant (*r^2^* = 0.50, *P < *0.001) (Fig. 3B). The *Firmicutes*/*Bacteroidetes* ratio (log_2_) (*r^2^* = 0.35, *P = *0.004), age (*r^2^* = 0.12, *P < *0.001) (Fig. 3C) and height (*r^2^* = 0.09, *P = *0.005) were significantly associated with PCoA ordination. Interindividual differences outweighed the diets, and most samples clustered by participant (PERMANOVA, *F *=* *6.38, *P < *0.001) (Fig. 3F).

**FIG 4:**
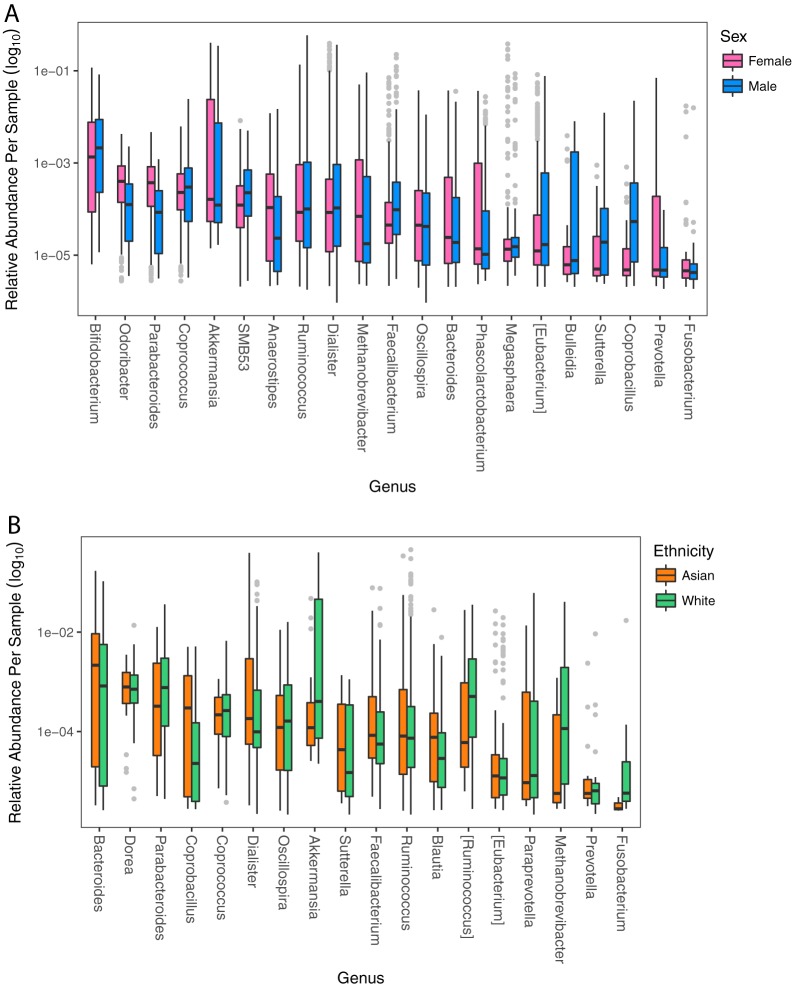
Differentially abundant OTUs between sex (A) and ethnicity (B) are displayed at the genus level. Significance was determined with DESeq2 and accounted for age, diet, diet order, saturated fat level, and sex or ethnicity.

Sex displayed significant grouping as assessed by nonparametric multivariate analysis of variance (PERMANOVA, *F *=* *4.33, *P < *0.001) (Fig. 3D). This test is sensitive to group dispersion and location within the ordination and therefore is able to identify if clustering occurred. Eighty-four OTUs were differentially abundant between the sexes on the baseline diet when adjusting for age and ethnicity (Fig. 4A), but there were no differences in alpha and beta diversity. There were no interaction effects between sex and saturated fat level or dietary protein source. Males had a significantly higher *Firmicutes*/*Bacteroidetes* ratio (log_2_) on both the baseline (ANOVA, *F *=* *14.97, *P < *0.0001) and experimental diets (three-way ANOVA, *F *=* *8.72, *P = *0.0039).

Ethnicity was another factor significantly influencing the ordination (PERMANOVA, *F *=* *4.75, *P < *0.001) (Fig. 3E). When comparing the three major groups of whites, Asians, and African Americans, alpha diversity (Shannon index) between groups was significantly different on both the baseline diet (ANOVA, *F *=* *4.76, *P = *0.0110) and the experimental diets (three-way ANOVA, *F *=* *4.60, *P = *0.0126). On the baseline diet, the greatest diversity was in African Americans (3.98 ± 0.36), then whites (3.70 ± 0.48), and lastly Asians (3.45 ± 0.55), and values were not significantly different compared to experimental diets. When comparing the two largest groups of whites and Asians on the baseline diet, there were 90 significantly different OTUs (Fig. 4B). The Human Microbiome Project data indicated that ethnicity correlated with various microbiome traits (34); however, the utility of American ethnicity has been questioned because it incorporates social/economic/cultural differences that cannot be separated from genetics in their influence on the microbiome (18). Therefore, we consider ethnicity a “meta-trait” that incorporates all of these mentioned factors.

### Individual traits are correlated with microbiome genera.

**FIG 5:**
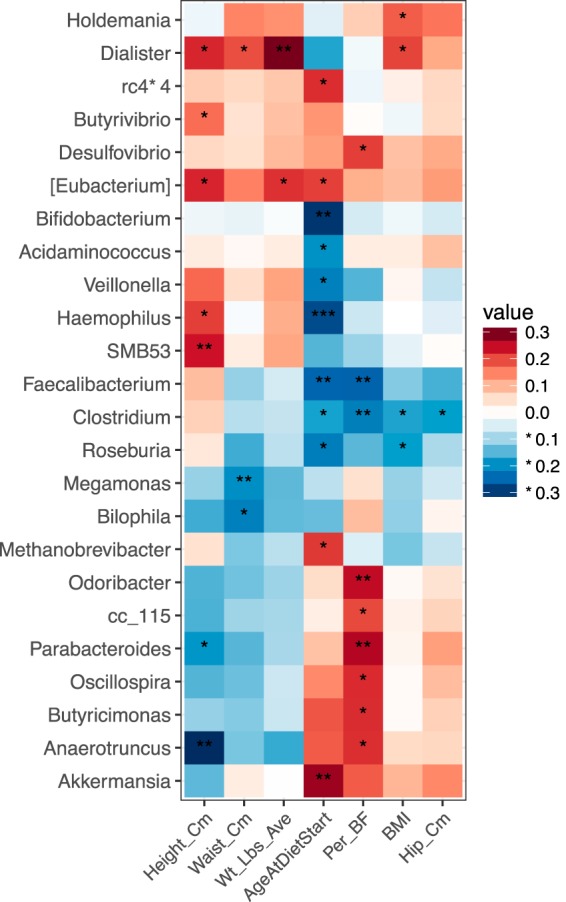
Microbiota are correlated with physical traits. Correlations between genera and traits were conducted using nonparametric Spearman correlation and organized into heat maps with hierarchical clustering. *P* values were determined by permuting by participant, and significant correlations are designated with asterisks representing *P* values where *** is <0.001, ** is 0.001 to 0.01, and * is 0.01 to 0.05.

Correlations between genera and individual traits were estimated using nonparametric Spearman correlation, and significance was estimated by permuting (*n* = 9,999) all four participant samples to account for nonindependence of samples taken from the same participant. Results were organized into heat maps with hierarchical clustering. Of the physical characteristics, the greatest number of significant correlations were with age (years) (11 genera), body fat (%) (9 genera), and height (cm) (7 genera), while waist (cm) and hip (cm) measurements had no significant results (Fig. 5). *Haemophilus* was most significantly related to age (rho = −0.28, *P = *0.0009), and then *Bifidobacterium* (rho = −0.31, *P = *0.0014), which is known to correlate strongly with age (15, 35). *Faecalibacterium* (rho = −0.26, *P = *0.007) and *Roseburia* (rho = −0.22, *P = *0.01) were also significant and found to be characteristic of older people (36), but *Akkermansia* (rho = 0.28, *P = *0.0028) and *Haemophilus* are novel associations with age because they have not been seen previously (15). Height has been observed to correlate with the microbiome (37), but mechanisms explaining this are unexplored. Percent body fat had many significant correlations, and the overall pattern was similar to that of age, but it was disparate from other body measurements like BMI and weight. The microbiome is known to play a role in obesity (20, 22, 38), but studies rarely report both body fat (%) and BMI.

### Protein source effect on the microbiome was masked by saturated fat level.

**FIG 6:**
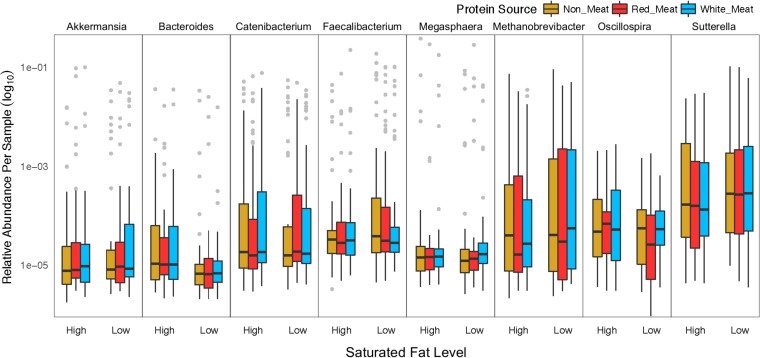
“Protein-sensitive” OTUs were determined to respond to any change in protein source regardless of saturated fat level. Differentially abundant OTUs between each protein diet were determined with DESeq2 and accounted for age, diet order, saturated fat level, sex, and ethnicity. OTUs that were differentially abundant in all comparisons of protein source were determined to be “protein sensitive.”

Dietary protein source influenced the microbiome, but it was apparent only once data were analyzed separately for high and low saturated fat intake. Once separated, many OTUs were determined to be differentially abundant with a greater number of differences found within the high-saturated-fat group (Table 1). The common number of OTUs between all three protein source comparisons within low-saturated-fat (76 OTUs) and high-saturated-fat (145 OTUs) groups was greater than half of the identified OTUs, which suggests that these microbes were responding to any change of protein source rather than a particular dietary protein. Of these common OTUs, 19 were consistent between the low and high saturated fat levels and were designated “protein-sensitive OTUs” because they responded regardless of saturated fat level (Fig. 6). Protein source has been shown to alter the microbiome composition, and *Bacteroides* and *Sutterella* were two commonly changed taxa between this study and one conducted with rats (39).

**TABLE 1:** Differentially abundant OTUs between protein sources

Diet comparison	No. of OTUs		
	All data	Saturated fat level	
		Low	High
Red meat vs nonmeat	3	115	203
White meat vs nonmeat	1	145	198
Red meat vs white meat	0	130	240

### Microbiome diversity influences response to experimental diet.

**FIG 7:**
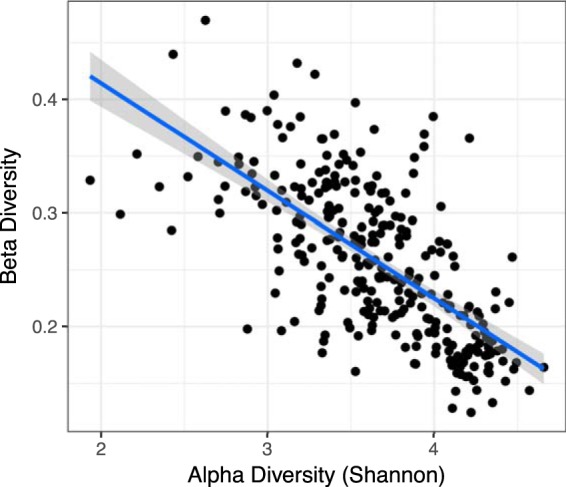
Alpha diversity predicts beta diversity. Linear mixed model using age, sex, ethnicity, saturated fat level, and protein diet as covariates was significant (*r*^2^ = 0.87, *P* value = 0.015). Beta diversity was calculated as the distance between the baseline diet and each experimental diet (three per person) for each participant.

Diversity is an important characteristic of a microbial community that has been associated with health status (40) and response to treatment (41). We found a strong negative relationship between alpha and beta diversity using a linear mixed model fit by restricted maximum likelihood where saturated fat level and protein diet were random effects and sex, ethnicity, and participant were fixed effects (*r *=* *0.88, *P = *0.0125) (Fig. 7). Higher alpha diversity predicted that communities changed less in response to the experimental diets, which has been observed previously in studies focused on starch and weight loss (42, 43).

## DISCUSSION

The experimental diets had a modest effect on the microbiome, and protein source was not as influential as saturated fat level. This is consistent with previous studies demonstrating that microbiota compositions are resistant to short-term interventions with long-term dietary patterns being the most influential (25, 27). The rapid changes observed in other studies may have resulted from comparing diets differing starkly in macronutrient ratio and food source (e.g., all plant or all animal) (31, 44). The experimental diets within this study were structured so the macronutrient ratios remained consistent and the levels created a diet sustainable for weeks. In addition, the focus was on altering nutrient sources of protein and saturated fat, not the critical fuel resource of microbial accessible carbohydrates (MACs) that can dramatically influence the microbiome (8). Due to a maintained macronutrient ratio and focus on nutrients that are not a central microbial resource, 4 weeks may not have been enough time to observe the less-direct effects of these dietary changes. In addition, small shifts may not have been captured by our taxonomic resolution because shifts from fiber were observed at the species level (45), which is a taxonomic level not well captured with our methods. This makes the differences that are seen very interesting.

Associations with interindividual differences outweighed the effects of the experimental diets. Many variables influenced the microbiome, and this highlights how sensitive the microbiome is to the accumulation of factors other than diet. In fact, two large-scale population studies identified 69 and 126 factors relating to interindividual and health traits that correlated with the microbiome (37, 46). This could help explain why it is common to see samples from a participant cluster together and for variability to be greater between individuals rather than within the same individual (9, 47). In addition, this difference in initial microbiome composition can influence the physiological effect of specific foods (29, 30) and the response to dietary interventions (9, 42).

There are many individual traits that can exert an effect on the microbiome. Sex differences within the microbiome are a debated topic because there are reports both supporting and refuting this notion, but it is likely that other variables such as diet, age, and genotype (14, 34, 48) are masking a real sex effect. When these sources of variability were controlled for in mice, there was a clear effect of sex on the microbiome that included diet interactions and was mediated, in part, by sex hormones (14). Obesity is another trait associated with the microbiome (22) where consistency has been questioned (38). Obesity is most commonly defined by BMI, but the calculation does not take into account body composition, and here, it appears body fat (%) has a stronger relationship with the microbiome. It is possible that the mechanism is related to levels of systemic inflammation because both body fat and aging (49) have been associated with increased inflammation, and inflammation has been correlated with microbiome changes (50–52). In fact, exceptionally healthy old Chinese individuals had similar microbiomes as healthy young people (53), suggesting that health status, not age, may be the most important. Aging has been associated with changing taxa, but diversity appears to be stable within adults (15, 16, 54) until about 80 years of age, when it starts to decrease (15). The range in this study was 21 to 65 years old, and alpha diversity did not significantly change. All of these traits appear to be important factors in shaping the microbiome.

One of the most important qualities of the microbiome is diversity, as it has been associated with metabolic and physiologic health, inflammation, and even response to inflammatory bowel disease therapy (55, 56). A common observation is that higher diversity is more beneficial, which follows the ecological theory that increased diversity provides greater functional resilience to perturbations. We observed that higher alpha diversity predicted less change in the microbiome in response to experimental diets. Similarly, when participants on a resistant starch and weight loss diet were stratified as responders or nonresponders, the nonresponders had higher diversity (43). In addition, dietary interventions were successful in increasing low gene richness and clinical phenotypes (42), further supporting the idea that decreased diversity is a less optimal state.

Although dietary effects were outweighed by other factors, surprisingly, saturated fat level had more of an effect than protein source. Bacteria can use protein directly as a nitrogen source while fat is not considered an energy resource (11). The strongest argument in support of this observation is that dietary fat requires oxygen to be metabolized and the gut microbiota is dominated by strict anaerobes (57). However, bacteria can break down polyunsaturated fatty acids, and intermediates are found in host tissues (58). It is also known that some fatty acids have antimicrobial properties (57, 59). While utilizing fat as an energy source may be atypical for bacteria, there may be other ways in which bacteria can interact with fat that lead to a microbial community response.

Levels of saturated fat and monounsaturated fat were both altered in this study, suggesting that the ratio of these fatty acids rather than the overall fat amount may affect the microbiome. This is important because in many studies microbial changes associated with high-fat diets were achieved by concurrently reducing the amount of carbohydrate/fiber in the diet (60, 61). This reduction in microbial substrate, along with an indifference to fat profile, may confound the relationship between gut microbiota and dietary fat. When the amount of fat was held constant but fat sources of palm, olive, and safflower oil were compared, community composition changed and increases in obesogenic traits were associated with high saturated fat (62, 63). It may be that specific compounds within the fat sources are responsible for these effects because linoleic acid and oleic acid supplementation reduced body weight and visceral fat mass along with microbial taxa (64, 65). Other possible influences are fat-soluble vitamins and polyphenols. In addition, the microbiome has been reported to indirectly affect host lipid metabolism through short-chain fatty acid production and bile acid regulation (66). The relationship between dietary fat and the microbiome is clearly complicated and still not fully understood.

In conclusion, saturated fat level had a modest effect on the microbiome and masked a slight effect of dietary protein source. Our findings suggest that fat profile should be a consideration in reference to the microbiome. The influence of interindividual differences was greater than that of dietary interventions, but it is likely that longer periods of intervention would be needed to observe more significant changes. Moreover, the experimental diets were not focused on the main microbial resource, carbohydrates. Taken together, our findings provide evidence that shorter-term moderate dietary changes lead to a modest response of the microbiome, and that the resistance to change increases with microbial diversity.

## MATERIALS AND METHODS

### Study design.

The present study is part of the larger *A*nimal and *P*lant *PRO*tein *a*nd *C*ardiovascular *H*ealth (APPROACH) study. It was conducted, with IRB approval, to determine the interacting effects of saturated fat level and protein source on markers of cardiovascular disease risk, using a standardized baseline diet and six experimental diets. The baseline diet reflected the macronutrient ratio of the average American diet (67), while the experimental diets had reduced carbohydrate and elevated protein levels chosen based on previously shown therapeutic benefits of changing macronutrient ratios on cardiovascular disease risk (68). Results relating cardiovascular disease traits to the microbiome are not discussed here.

All participants (*n* = 109) first consumed a 2-week baseline diet and then three experimental diets in a split-plot design. They were randomly assigned to either low (7%E)- or high (15%E)-saturated-fat groups (Fig. 1). Fat level differences were created by altering amounts of high-fat dairy and butter, and only 2% to 3%E came from lean meat or tropical oils when on the nonmeat diet. Within each fat group, participants consumed, in random order, three isocaloric diets with 12%E derived from different protein sources: nonmeat (legumes, nuts, grains, isoflavone-free soy products), lean cuts of red meat (11%E beef, 1%E pork), or white meat (8%E chicken, 4%E turkey). The remaining protein source in all diets (13%E) consisted of eggs, dairy, and vegetable protein. All fish, seafood, and processed meats were excluded from the diets, and grain-finished beef was used because it comprises 96% of the U.S. beef market (69). Each experimental diet was consumed for 4 weeks, with a two-week, but up to seven-week, washout period between diets where participants ate their regular home diets (Fig. 1). Experimental diets were prepared by the Bionutrition Unit of the University of California, San Francisco Clinical and Translational Studies Institute using 4-day rotating menus. Dietary compliance was determined during the second week of the baseline diet and third week of each experimental diet by measuring 24-h urinary nitrogen and creatinine levels (Quest Diagnostics). Fecal samples were collected at the completion of all four diets and kept frozen until analyzed. Further details can be found in the work of N. Bergeron, S. Chiu, P. T. Williams, S. King, and R. M. Krauss (submitted for publication).

Samples were available from 109 participants, and ethnicity was self reported using categories of white (*n* = 60), Asian (*n* = 20), African American (*n* = 13), Native American (*n* = 1), Pacific Islander (*n* = 1), white/Native American (*n* = 6), white/African American/Native American (*n* = 2), or unreported (*n* = 6). For analyses, all reported groups except white, Asian, and African American were combined to form an “other” category (*n* = 10).

### DNA extraction, library preparation, and sequencing.

Microbial DNA extraction and sequencing were adapted from the methods developed for the NIH-Human Microbiome Project (34). DNA was extracted from human feces using a MoBio Power Soil DNA extraction kit (MoBio, Carlsbad, CA). DNA of the V4 hypervariable region of the 16S rRNA gene was amplified with barcoded primers (515f and 806r [70]) in triplicate using the 5 PRIME HotMasterMix (VWR). Products were quantified with Quant-iT PicoGreen dsDNA assay kit (Thermo Fisher), and samples were combined in equal amounts (∼250 ng per sample) to be purified with the UltraClean PCR cleanup kit (Mo Bio). Pooled amplicons were sequenced on the Illumina HiSeq 2500 platform over two lanes to generate single-end reads. Postquality filtering and removing OTUs representing <0.005% of all OTUs to reduce the sparsity of the data set generated 109,811,869 total reads, with an average of 255,906 reads per sample. Nineteen samples had less than 1,000 reads and were removed, as they were considered unsuccessfully sequenced.

16S microbial data were processed using QIIME version 1.9.1 (71). Barcodes were matched to FASTQ files and then removed (72). Similar sequences (97%) were combined into operational taxonomic units (OTU) using open picking (73) with SUMACLUST (74, 75). Representative sequences for each OTU were aligned using PyNAST (76). The lanemaskPH was used to screen out the hypervariable regions, and OTUs were classified with the Greengenes database (77). Samples were rarefied (78, 79) to a depth of 74,457, which removed three samples and resulted in a total of 410 samples used for analyses.

### Statistical analyses.

Microbiome communities were visualized using unweighted UniFrac (80) with principal-coordinate analysis (PCoA) using the phyloseq package (81). Differences among groups were tested using nonparametric multivariate analysis of variance (PERMANOVA) (82). Continuous variables were fit to the PCoA ordination by regression using the envfit function in the vegan R package (83), and *P* values were determined using 999 permutations. Beta diversity representing microbiome change in response to the experimental diets was calculated as the distance between the experimental diets and baseline diet in ordination space. Alpha diversity was assessed using the Shannon diversity index, which takes into account richness and evenness, that is, if few taxa dominate the community or many taxa are evenly represented. Analysis of variance (ANOVA) with Tukey *post hoc* tests to correct for multiple comparisons was used to detect significant differences in measured traits. Mixed models using a fixed effect for each participant were included when appropriate. Differential abundance was determined on nonrarefied data normalized by size factors estimated by the median-of-ratios method using a negative binomial Wald test that uses standard maximum likelihood estimates for generalized linear model coefficients. *P* values were corrected for multiple comparisons using the Benjamini-Hochberg method, and alpha was set to 0.01 using the DESeq2 R package (84) on nonrarefied data as suggested (78, 79). Correlations between genera and traits were estimated using nonparametric Spearman correlation, and *P* values were estimated by permuting (*n* = 9,999) all four participant samples to account for nonindependence using the permute R package (85). Samples for correlations (*n* = 344) were included from only participants (*n* = 86) who had all four samples to allow for permutations. All analyses were conducted in R v 3.3.2.

### Data accessibility.

Sequencing data have been deposited in the NCBI Sequence Read Archive under the accession number PRJNA498128.
